# Strengthening community and stakeholder participation in the implementation of integrated vector management for malaria control in western Kenya: a case study

**DOI:** 10.1186/s12936-021-03692-4

**Published:** 2021-03-19

**Authors:** Peter N. Ng’ang’a, Polycarp Aduogo, Clifford M. Mutero

**Affiliations:** 1 International Centre of Insect Physiology and Ecology (ICIPE), PO Box 30772, Nairobi, Kenya; 2 School of Public Health, Jomo Kenyatta University of Agriculture and Technology, PO Box 62000, Nairobi, Kenya; 3 University of Pretoria Institute for Sustainable Malaria Control (UP ISMC), School of Health Systems and Public Health, University of Pretoria, Private Bag X363, Pretoria, 0001 South Africa

**Keywords:** Malaria, Community participation, IVM, Advocacy, Social mobilization, Capacity building

## Abstract

**Background:**

Integrated vector management (IVM) is defined as a rational decision-making process for the optimal use of resources for vector control. The IVM approach is based on the premise that effective control of vectors and the diseases they transmit is not the sole preserve of the health sector. It requires the collaboration and participation of communities and other stakeholders in public and private sectors. Community participation is key to the success of IVM implementation at the local level.

**Case description:**

The study was conducted in Nyabondo, a rural area of Kenya where malaria is endemic. The objective of the project was to promote adoption and sustainability of IVM and scale up IVM-related activities as well as increase community participation and partnership in malaria control through outreach, capacity-building and collaboration with other stakeholders in the area. Collaboration was pursued through forging partnership with various government departments and ministries, particularly the fisheries department, ministry of education, ministry of health, forestry department and the social services. In total, 33 community-based organizations working within the area were identified and their role documented. Through distribution of information, education and communication (IEC) materials alone, the project was able to reach 10,670 people using various social mobilization methods, such as convening of sensitization meetings—dubbed 'mosquito days'—mainly spearheaded by primary school pupils. A total of 23 local primary schools participated in creating awareness on malaria prevention and control during the project phase. The collaboration with other departments like fisheries led to stocking of more than 20 fishponds with a total of 18,000 fingerlings in the years 2017 and 2018. Fish ponds provided an opportunity for income generation to the community. In partnership with the county government health department, the project was able to re-train 40 CHVs on IVM and malaria case management in the area. Additionally, 40 fish farmers were re-trained on fish farming as part of income generating activity (IGA) while 10 CBOs made up of 509 members received both eucalyptus and *Ocimum kilimandscharicum* seedlings that were distributed to four CBOs composed of 152 members. Four primary schools made up of 113 health club members also received eucalyptus seedlings as part of IGA in addition to fish farming. In total, around 20,000 eucalyptus seedlings were distributed to the community as part of IGA initiatives. By the end of 2018, the project was able to reach 25,322 people in the community during its two-year advocacy and social mobilization initiatives.

**Conclusion:**

Through advocacy and social mobilization, the IVM strategy improved inter-sectoral collaboration, enhanced capacity building and community participation. However, more IVM related activities are needed to effectively mobilize available resources and increase community participation in malaria control.

## Background

Globally, malaria is a life-threatening parasitic disease which has persisted as a major public health problem. In 2019, an estimated 229 million cases of malaria occurred worldwide resulting in 409,000 deaths [1]. The World Health Organization (WHO) African region accounted for 94% of all malaria deaths in 2018 [2]. Children aged under 5 years are the most vulnerable group affected by malaria. In 2018, children below five years accounted for 67% (272 000) of all malaria deaths worldwide [2]. The consequences of such high rates of malaria go far beyond health and affect the socio-economic circumstances and wellbeing of the affected communities and countries in which malaria is endemic. Malaria is known to contribute to poverty in African households as a result of labour days lost due to the illness caused [3]. It also directly impacts households due to the costs incurred towards treatment and caring for those who fall sick [4, 5]. In Africa, poverty also contributes to increased malaria risks as people live in poor housing conditions that expose them to mosquito bites [6–9]. More importantly, such people also have inadequate access to health services required for prompt diagnosis and effective treatment of the disease [10].

Community participation plays a vital role in malaria prevention and control especially at community level [11–13]. Participation promotes self-awareness and confidence and causes people to examine their problems and to think positively about the available solutions. It also increases the sense of control over issues that affect the lives of community members [14]. Community participation also serves as an instrument for empowering people and facilitating better living conditions for those who are underprivileged within the society [13, 15, 16].

In most countries, vector control programmes commonly focus on a single disease whose prevention and treatment is primarily viewed as being the sole responsibility of the ministry of health. Thus, sectors, such as agriculture, construction, local government, environment, construction, fisheries and tourism, are often insufficiently aware of the consequences of their actions on the incidence of vector-borne diseases, or the potential opportunities within the sectors to address such challenges [17]. In order to correct this anomaly, the WHO recommends integrated vector management (IVM) as a means of rationalizing and subsequently optimizing the use of the available resources for vector control [18, 19]. The WHO has identified evidence-based decision-making, collaboration within the health sector and with other sectors, as well as advocacy, social mobilization, legislation and capacity-building as the key elements of IVM [18, 20]. IVM also incorporates interventions, actors and resources coordinated between the health and other sectors, including communities, the private sector and non-health ministries, such as agriculture and housing [18]. The approach seeks to improve the efficacy, cost-effectiveness, ecological soundness and sustainability of disease-vector control and limit the indiscriminate use of insecticides, based on a thorough understanding of local ecological conditions [18, 20, 21].

A report by Chanda et al*.* in Zambia [22], described a comprehensive and successful IVM program that has been implemented by the Zambian National Malaria Control Programme. Over a relatively short time, this programme expanded coverage of vector control interventions, leveraged additional resources and successfully reduced malaria-related morbidity and mortality. While much remains unknown about the impact of fully developed IVM programmes on malaria transmission, the IVM approach continues to be a strategy with great promise for disease control in Africa [23–26]. Historically, programmes with IVM elements have brought about significant reductions in vector populations and malaria transmission across a range of transmission settings [25, 27–29]. "Community based" participatory IVM projects incorporating larval source management, habitat manipulation and multi stakeholder engagement have been implemented in different settings in Western Kenya [29–31], as well as in Dar es Salaam, Tanzania and Botor-Tolay District, Oromia regional state of Southwestern Ethiopia [32, 33].

Advocacy is a process that uses communication to influence decision making as performed by the decision makers. Most often, advocacy uses interpersonal communication at events and meetings to inform decision-makers about a problem [34]. Advocacy activities make the case for specific causes which may include changes in malaria-related policies (requiring actions by high level officials or groups), improvements in funding, or increased priority and political will [35]. The approaches used in advocacy are very similar to the approaches used in behaviour change communication–interpersonal communication, mass media such as newspapers and printed materials. Social and behaviour change communication targets individuals at household levels or service providers. This type of communication targets the behaviours, habits and practices of the populations in relation to their health [34, 36].

In Kenya, potential challenges hindering malaria advocacy, communication and social mobilization (ACSM) initiatives include weak inter-sectoral collaboration, low funding and prioritization of malaria ACSM activities, poor delivery of ACSM interventions in counties and poor allocation of limited resources for malaria at the community level [37]. Other challenges includes lack of proper coordination and duplication of partner activities, over-reliance by county governments on funding from the National Malaria Control Programme for ACSM activities, ambiguity in the devolution process regarding roles and responsibilities of county and national governments with regard to malaria control, misappropriation of funds, poor integration of health and non-health sectors resulting in low leverage of ACSM resources amongst partners [37].

This paper present a project report on a participatory integrated vector management (IVM) project implemented to mitigate malaria in a rural area, western Kenya, from 2016 to 2018. The overall aim of the project was to reduce malaria and improve health and livelihoods of the local community through a range of health, social and economic outreach and capacity-building activities. The report is intended to share practical field experiences, challenges, achievements and lessons relevant for development, promotion, and adoption of IVM-ACSM strategies for malaria control in high-transmission endemic areas in sub-Saharan Africa.

### Intervention area

The project was carried out in Nyabondo, a plateau area located in Upper Nyakach, Kisumu County, about 30 km North-East of Lake Victoria. Nyabondo lies between an altitude of 1520 m and 1658 m above sea level, and 0° 23′ 0 S and 34° 58′ 60 E. The area is host to an estimated 34,000 people with a high population density of nearly 460 persons per square kilometre (km) [38]. The community largely depends on brick making and small scale mixed farming activities, such as crop/fish farming and livestock keeping as the main economic activities [39]. Agricultural activities are dominated by crops such as maize, cassava, sorghum and sweet potatoes [29, 40]. Malaria endemicity in this region is characterized by perennial parasite transmission by *Anopheles* vectors due to favourable environmental and climatic conditions particularly rainfall, temperature and humidity [37, 41]. The overall average malaria parasite prevalence by 2015 was about 27% for the Lake Victoria Basin [37] and the recorded overall average malaria prevalence among school children is 32.4%, which is highest compared to other parts of Kenya [42]. Previous entomological surveys in Nyabondo found that larval *Anopheles* mosquitoes bred in both temporary and permanent habitats with *Anopheles arabiensis* being the main malaria vector species (99.3%), followed by *Anopheles gambiae* (0.7%) [43]. It has been observed that abandoned ponds were more preferred breeding habitats for *Anopheles* malaria vectors when compared to other potential habitats [40]. Being a rural setup, the overall poverty incidence in Nyabondo was around 40.1% in 2016 [44]. The project area has been described previously in a KAP survey on malaria prevention and house screening by Ng’ang’a et al*.* [39].

### Project design

Mixed methods of data collection were applied where quantitative and qualitative approaches were used during the project phase. Baseline surveys were conducted to gather background information on the community knowledge, attitudes and practices on house screening, malaria prevention and control [39].

### Baseline surveys

#### Knowledge and perceptions on malaria prevention and house screening survey

A baseline cross-sectional household survey was conducted in January 2017. Structured questionnaires and focus group discussions [FGDs] were used to collect data on; (i) community knowledge on malaria prevention, (ii) use of personal protection measures against malaria and mosquito bites, (iii) perceived benefit for screening doors, windows and eaves.

### LLINs ownership, coverage and utilization survey

Another cross-sectional household survey was conducted in November 2017 with an objective of assessing LLINs ownership and utilization in the area. Data was collected using structured questionnaires and focus group discussions. The questionnaires and FGD questions focused on various sub-themes like; knowledge on signs and symptoms of malaria, knowledge of mosquito breeding places, malaria prevention and control, LLINs ownership and use, LLNs acquisition, their condition, and the household perceptions on LLINs use.

#### Advocacy and social mobilization

Local people were sensitized on integrated approaches for malaria control. This was done by using appropriate information, education and communication (IEC) materials that were developed by the project. The project enhanced knowledge and skills through door to door education and also through conducting various trainings. IEC/BCC materials were distributed using various channels. Primary schools participated in dissemination of malaria control information during various forums like the World Malaria Days and during locally organized mosquito day celebrations. The primary schools got engaged in various malaria dissemination activities involving drama, dances, health talks, plays /songs, malaria posters presentations and role plays. All these malaria dissemination activities had different themes on symptoms and signs of malaria, knowledge of mosquito breeding places, malaria prevention and control, LLNs use, and community’s perceptions on malaria control.

#### Inter sectoral collaboration

The project forged partnership with various government ministries. The ministries and departments involved included: the ministries of health; agriculture; and forestry. Furthermore, the fisheries department was involved in fish farming while ministry of education was involved in establishing and strengthening of school health clubs. The primary school health clubs were made up of pupils, with the teacher in-charge acting as the patron. The Ministry of Health (MoH) was involved in distribution of LLINs, training of CHVs and monitoring of malaria in the community.

#### Capacity building

Several community based trainings were carried out during the project phase. The objective was to build capacity for malaria control and resource mobilization. The project managed to: train CHVs on IVM and malaria case management; conduct refresher training for the fish farmers; train the local youth group (MOCON) on house eaves screening; and train the field project staff on the application of an android-based data collection tool known as ‘Zzapp Malaria’—a map-based malaria elimination app [45], aimed at improving the tracking of mosquito breeding sites, in order to accurately target them with the application of biolarvicides, such as *Bacillus thuringiensis* *var. israelensis (Bti)* for the control of mosquito larvae.

## Results

### Knowledge and perceptions on malaria prevention and house screening survey

Ninety one percent (91.3%) of the respondents (n = 80) reported to have previously received or heard malaria related messages in the area. Two leading reported sources of malaria information in the area were the International Centre of Insect Physiology and Ecology (ICIPE) (87.5%) and Radio (62.5%). Government health department was mentioned by 37.5% of the respondents for its involvement in the distribution of mosquito nets and awareness creation. Ministry of Health was also recognized for its involvement in health education and promotion initiatives mainly through the community health volunteers [CHVs]. Concerning malaria information and communication messages, use of mosquito nets was by far mentioned as the most recalled or widely known personal protection method (reported by 94.7% of the respondents).

However, despite reported high level of awareness and use of insecticide treated mosquito nets (97.4% and 97.6% respectively), only 15.6% of the respondents were aware of screening of windows, eaves and doors as a protection method against malaria. About 58% of the respondents acknowledged not having heard any information or knowledge on house screening. Most of the respondents reported that screening doors, windows and eaves would prevent entry of mosquito and other insects into their houses (> 85%). All the 80 (100%) respondents who participated in this survey were willing to screen their houses if facilitated and given information on how to screen while 96.3% of the respondents were willing to participate and collaborate in future IVM activities in the community. Detailed information on this survey is available in a published work by Ng’ang’a et al. [39].

### LLINs ownership, coverage and utilization survey

During this cross-sectional household survey, 65% of respondents were females and 35% were males [n = 160]. Commonly cited signs and symptoms of malaria were fever (24.1%), headache (17.7%), vomiting (14.5%) feeling cold (12.6%) and loss of appetite (10%). Children under five years were reported to be at higher risks of malaria infection (28.6%), followed by all children (22.2%), adult men (17.2%) and adult women (15.8%). Use of treated mosquito nets was reported as the most known and applied method for personal protection at household level (96%). Other common personal protection methods were rarely known and applied in the community. Knowledge of personal protection methods significantly varied based on the village of origin (χ2 = 302.101; df 112; P = 0.000, 95% CI), as well as education level of respondents (χ2 = 87.555; df 35; P = 0.000, 95% CI).

In total, there were 382 reported LLINs among the 753 occupants of the surveyed households [n = 160]. The overall average LLIN ownership per household was 2.4 (382/160) with 1.97 (753/382) persons per LLIN (calculated in households that owned nets). Around ninety seven percent (96.9%) of households owned at least one LLIN and 64.1% owned at least one LLIN for two people. Most households owned more than one LLIN, with 75% owning between 2–3 LLINs per household. LLINs ownership per household ranged from 0 to 6 with a mean of 2.39 and a median and mode of two each. Two of the most reported benefits of using LLINs in the study area were protection of the household members from getting bitten by indoor mosquitoes (44.1%), and protection from getting malaria (43.4%). Nets were also reported to offer protection against bites from other indoor nuisance insects (8.6%), (n = 290). Majority of the nets (80%) were reported to have been acquired less than six months before the interview time with 25% having been acquired less than three months prior to the survey period. Additional information on impacts of LLINs in comparison with other interventions in the project area is available in a published document by Mutero et al*.* [42].

### Stakeholders mapping and engagements

Active exploration of community structures and networks were undertaken during the project phase. In total, 33 community based organizations (CBOs) working within Nyabondo were identified and their role in vector control documented. A CBOs networking forum was held in early February 2017 to enhance partnership and collaboration towards sustaining IVM and by the end of that year, 16 stakeholders’ meetings were held with a total participation of 321 people (192 males & 129 females). Linkages were fostered through holding discussions around the agenda of working together and the need for intra- and inter-sectoral collaboration between the health and other sectors in controlling and sustaining malaria in the area.

### Social mobilization among community members and primary school students

**Fig. 1:**
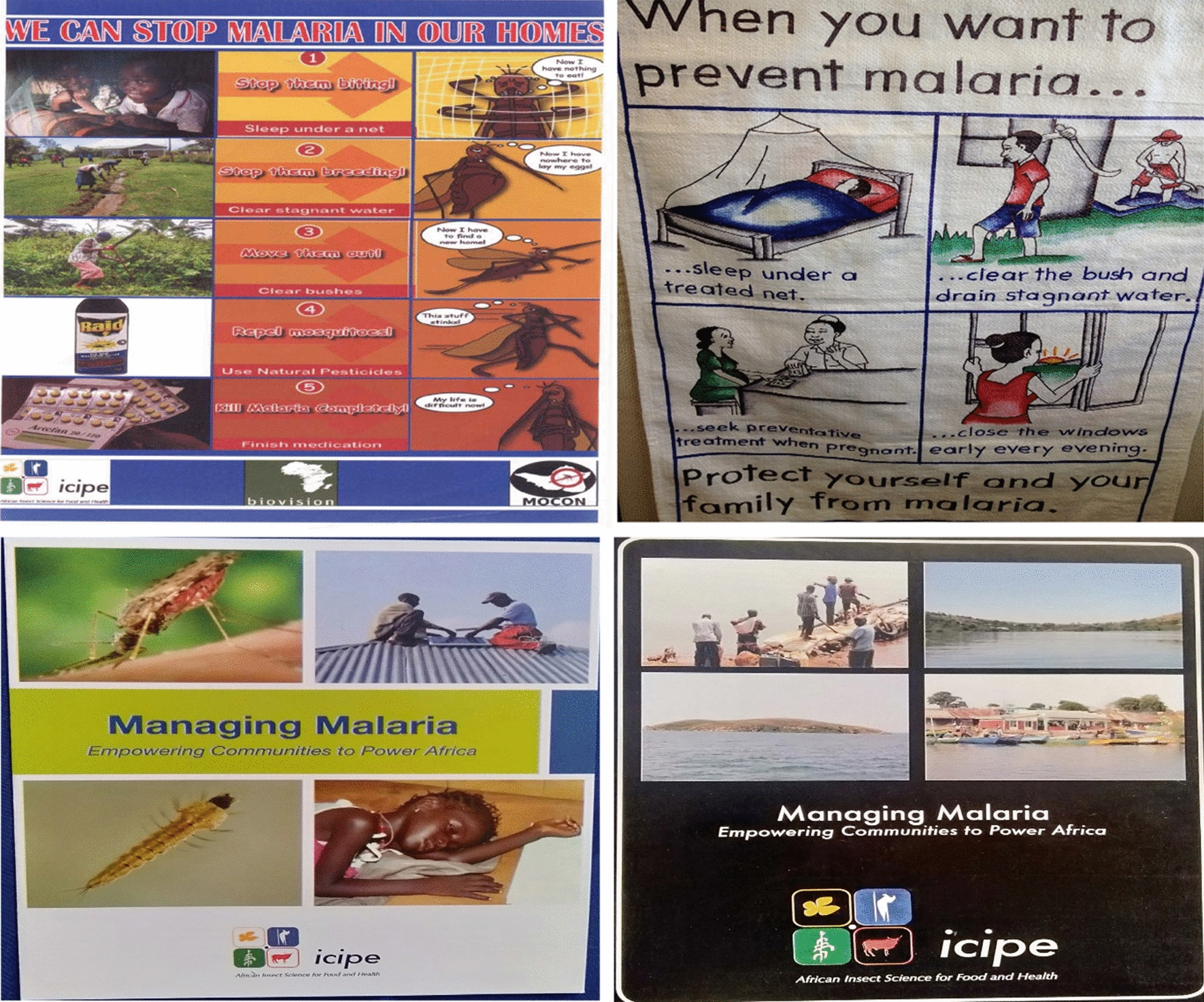
IEC materials distributed in the field to create awareness on malaria prevention and control. The last two (managing malaria) are ICIPE brochures distributed during stakeholders meeting and workshops

Social mobilization outreach activities began with baseline assessments that identified the levels of knowledge, attitudes and practices. Local people were sensitized on integrated approaches for malaria prevention. This was done using various information, education and communication (IEC) materials, such as posters, brochures and fliers (Fig. 1), in addition to holding health talks in schools. A primary school curriculum was developed and distributed to all schools through their health club patrons, who were mainly teachers in their respective schools.

The project enhanced knowledge and skills through door to door education and also through conducting various trainings in the community. IEC/BCC materials containing malaria messages continued to be distributed during community-based events such as mosquito days and through school health clubs. Information messages were prepared and got distributed with an aim of helping people understand the causes, transmission, prevention, control and health effects of malaria. Health education was also conducted in order to change community’s attitudes and behaviour towards malaria as well as enhance cooperation on malaria control. Distribution networks of IEC materials included use o:f local canteen patronized mainly by brick makers; chief’s Barazas (community meetings); primary school visits; locally organized farmers field days; CBOs networking forums; home/field visits; stakeholders meetings/forums; and use of other public forums particularly the internationally commemorated world malaria days and locally initiated mosquito days. Awareness was also created through publication and circulation of Mosquito Control in Nyabondo (MOCON) Bulletin. Through door to door education involving CHVs and project's field staff, the project managed to reach 800 households, (with estimated 4,000 occupants) with IEC materials in 2018.

The MOCON bulletins had draft articles prepared on various topics including; malaria control, cultivation of *Ocimum* plants and planting of eucalyptus trees to reclaim water-logged areas and produce wood for cooking as well as construction. Draft copies of the bulletin used to be prepared by the project field team and were submitted to ICIPE headquarters in Nairobi for review, editing and production. Upon publication, the copies were distributed in the project area using the local human resources which ensured that many people were reached with information on malaria prevention and control. As a result of this, over 2500 MOCON bulletin copies were distributed by the end of November 2018. In addition to this, the community also benefited from distribution of 300 braded T-shirts during the world mosquito days. The braded T-shirts displayed simple messages on malaria control and prevention. Through various distribution networks**,** 5094 females and 5576 males were reached with IEC/BCC materials during different events.

**Fig. 2:**
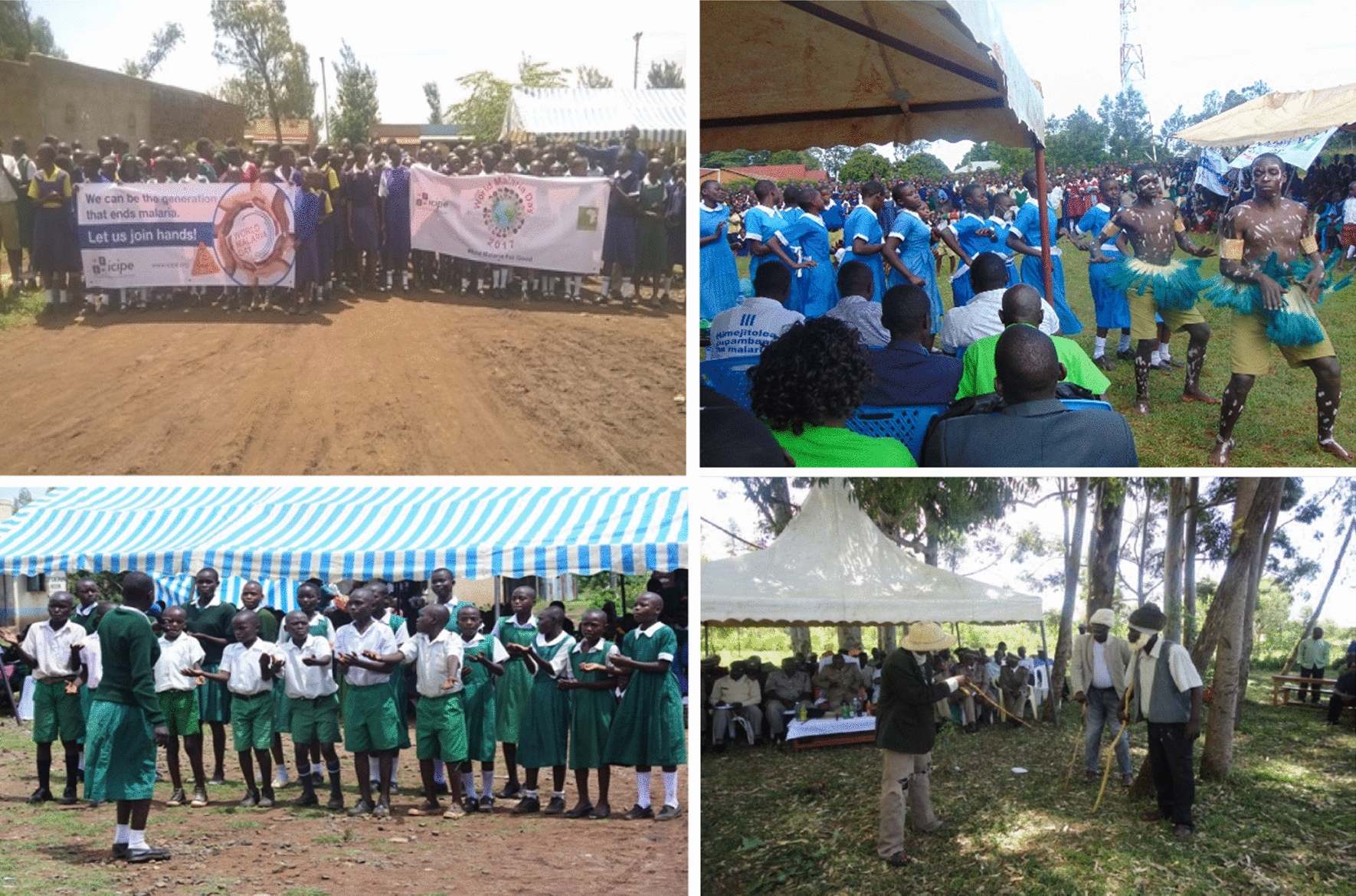
Malaria control procession during world malaria day in 2017 (Upperleft). Malaria related dances & poems (Upper right & lower left) and a local youth group performing a drama on malaria prevention & control (Last picture)

**Fig. 3:**
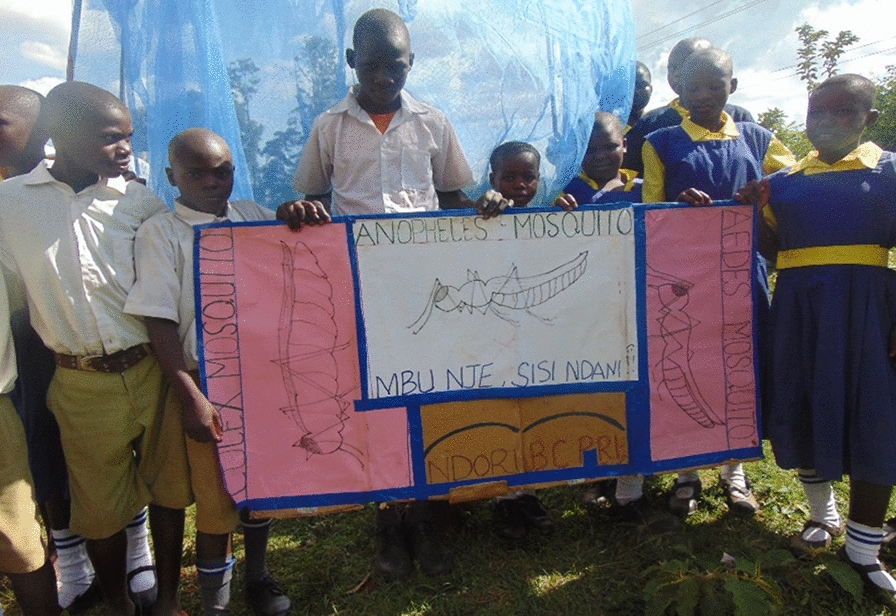
Pupils displaying messages about malaria vectors and demostrating LLINs use (at the back) during mosquito day

Several school consultative meetings were held in the area with the participation of 23 primary school health club patrons. The meetings aimed at planning on how to use primary school pupils in creating awareness on malaria prevention and control in the community. The primary school pupils participated in disseminating information on malaria control and prevention during various forums like the World Malaria Day and locally organized mosquito day celebrations in 2017–2018 (Fig. 2). Various schools got involved in drama, dances, health talks, plays/songs, malaria posters presentations and role plays, all of which covered various themes on malaria. Messages communicated were on symptoms and signs of malaria, knowledge of mosquito breeding places, malaria prevention and control, LLNs use, and community’s perceptions on malaria control (Fig. 3). The best schools were rewarded with mosquito nets and project branded T-shirts as recognition of their active participation in awareness creation.

### IVM Advocacy at policy making level

A multi-county IVM workshop was conducted in July 2018 at ICIPE’s Thomas Odhiambo Campus (ITOC), located at Mbita Point on the shores of Lake Victoria, western Kenya. Participants were drawn from six Lake Victoria Basin counties namely Kisumu, Siaya, Migori, Vihiga, Homa Bay and Kisii and also from the National Malaria Control Programme (NMCP), the national Vector-Borne Disease Control Unit and Kenya Medical Research Institute (KEMRI). In total 40 participants representing various organizations, departments and institutions attended the workshop. The objectives of the workshop were: to advocate and raise awareness on IVM and its role in the control of malaria and other vector borne diseases; to identify opportunities for multi-sectoral collaboration in IVM; and, to develop a work plan for advocacy and scaling-out of participatory IVM approaches by county governments.

### Inter sectoral collaboration

Collaboration between the health and other sectors was enhanced throughout the project phase. The institutional partnership was forged by engaging various government ministries and departments including Ministry of Agriculture (forestry department) for advice on income generation through agro-forestry farming activities; Fisheries department for fish farming; Ministry of Education for guidance on primary schools engagements; Ministry of Health for the distribution of LLINs, training of CHVs and monitoring of malaria prevalence; and department of social services which was the official umbrella for all community-based organizations (CBOs) working in the area. The collaboration with the fisheries department led to a stocking of more than 20 fishponds with a capacity to accommodate 18,000 fingerlings between 2017 and 2018. In total, 4800 mature table size fish were harvested in April 2018 and sold out at an average of 120 Ksh. (1.2 US$) each in local institutions like schools and hospitals. This resulted into an estimated fish sale amounting to Ksh. 576,000 or 5760 US$ during this period. This was expected to enhance/supplement household income for the pond owners.

### Capacity building for malaria control

**Fig. 4:**
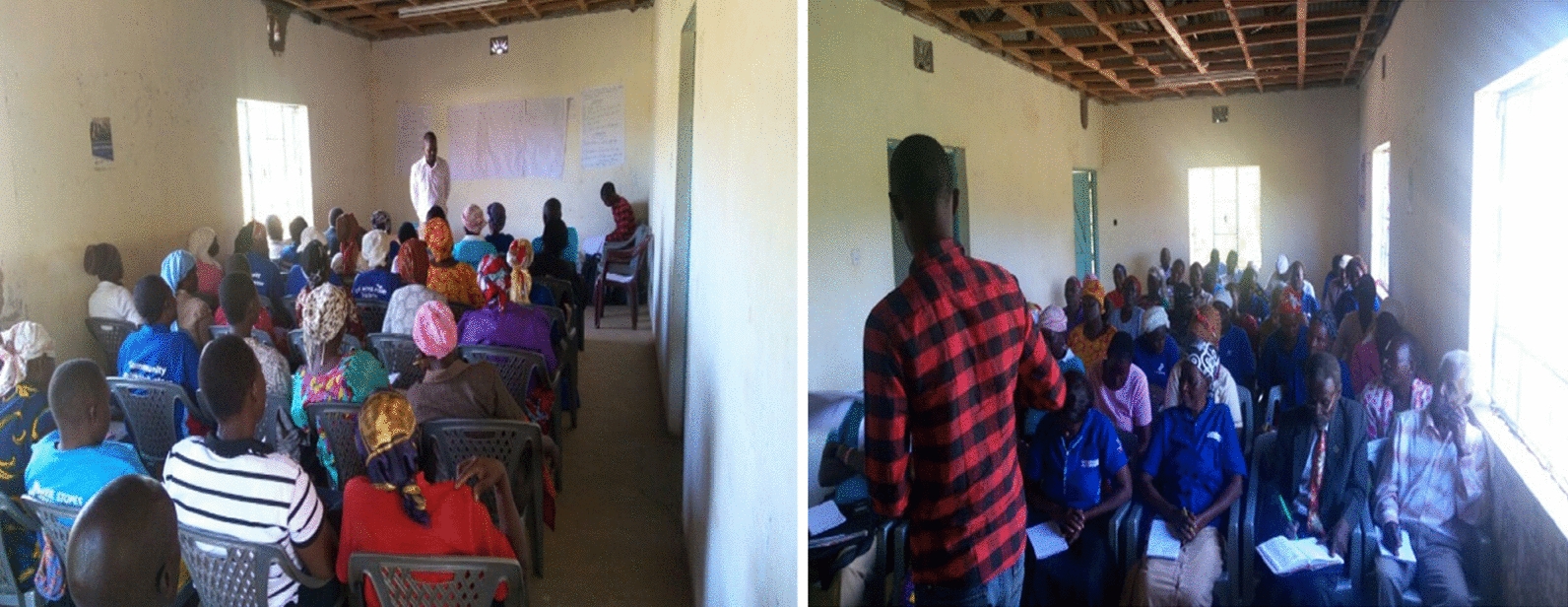
Ongoing training sessions for CHVs in the field

Through consultation and partnership with the county government, department of health, the project was able to train 40 CHVs on IVM and community case management of malaria. Before the training, several preparatory meetings were held to plan and work out the methodology and strategy for the training. The two teams agreed upon the training dates, venue, the training materials and the key contents for the training. The project financed and facilitated the training locally while the Ministry of Health provide the human resources and conducted the training. A CHV training manual/guide was jointly developed prior to the training date. Finally, a joint CHV training was conducted in 2018 where 40 CHVs were trained for three consecutive days (Fig. 4). The training topics included: community case management of malaria; testing and diagnosis for malaria using RDTs; malaria referral; keeping malaria records and reporting; basics of IVM (meaning, principles and elements); mosquito life cycle; disease transmission; and selection and application of vector control methods. The themes for the training were developed and prepared in line with the country’s community strategy guideline i.e. the Ministry of Mealth/CHV training manual/guide.

Selected CHVs were trained on integrated approach to malaria control so that they could promote social behavioural change in their day to day health related activities in the community. The training was a key exit and sustainability strategy for the IVM project. Among the 40 trained CHVs, six of them were hired by the project for around one year and together with a youth group, MOCON, they were tasked with responsibility of reaching out to the community with key IEC messages through; door to door education, community mobilization and sensitization. Before the end of the project in December 2018, three training guides/manuals were developed- i.e. CHVs training guide, fish farmers training manual and *Ocimum* farming training manual.

**Fig. 5:**
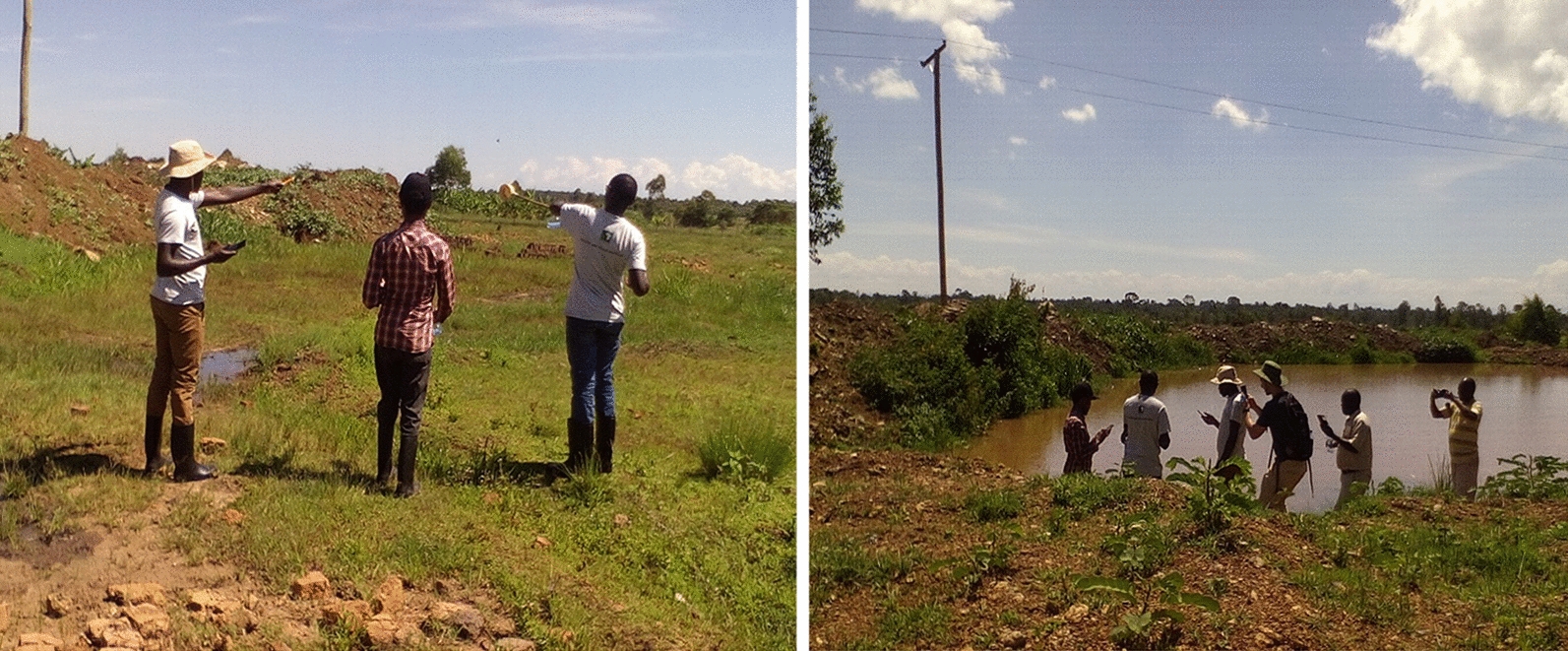
Field staff navigating through in the field to locate larval breeding sites using their mobile phones installed with Zzapp malaria software

As part of capacity building, five project staff were trained for five days in June 2018 on the application of Zzapp's map-based mobile app with an aim of guiding them in their daily  malaria control activities while streaming all field data to an interactive online dashboard. The data collection app was specifically meant to: reduce tedious paperwork in the field, enhance the efficiency of targeting mosquito larval habitats in the field and improve on methodology and capacity for monitoring larval populations by ensuring that all targeted mosquito breeding habitats are easily identified and treated with bio-larvicides in the field (Fig. 5).

Through a series of consultative meetings with the fisheries department, the project and the county government managed to conduct a day training for the fish farmers. Themes for the training were agreed upon after conducting a needs assessment involving community memmbers, the project team and the county government officials. The fish farmers training was attended by 23 males and 7 females. Other participants were representatives from Kenya Fisheries Research Institute (KEFRI), department of fisheries Kisumu County, Maseno University, ICIPE and MOCON. During this training, participants were informed about the potential of tilapia fish (*Oreochromis niloticus)* in biological control of mosquito larvae. Participants were also informed that once ponds are stocked with tilapia and properly managed they are able to generate income and improve nutrition for the households. It was expected that the accrued incomes or earnings generated from sales of seedlings and harvested fish would increase/ enhance household incomes.

**Fig. 6:**
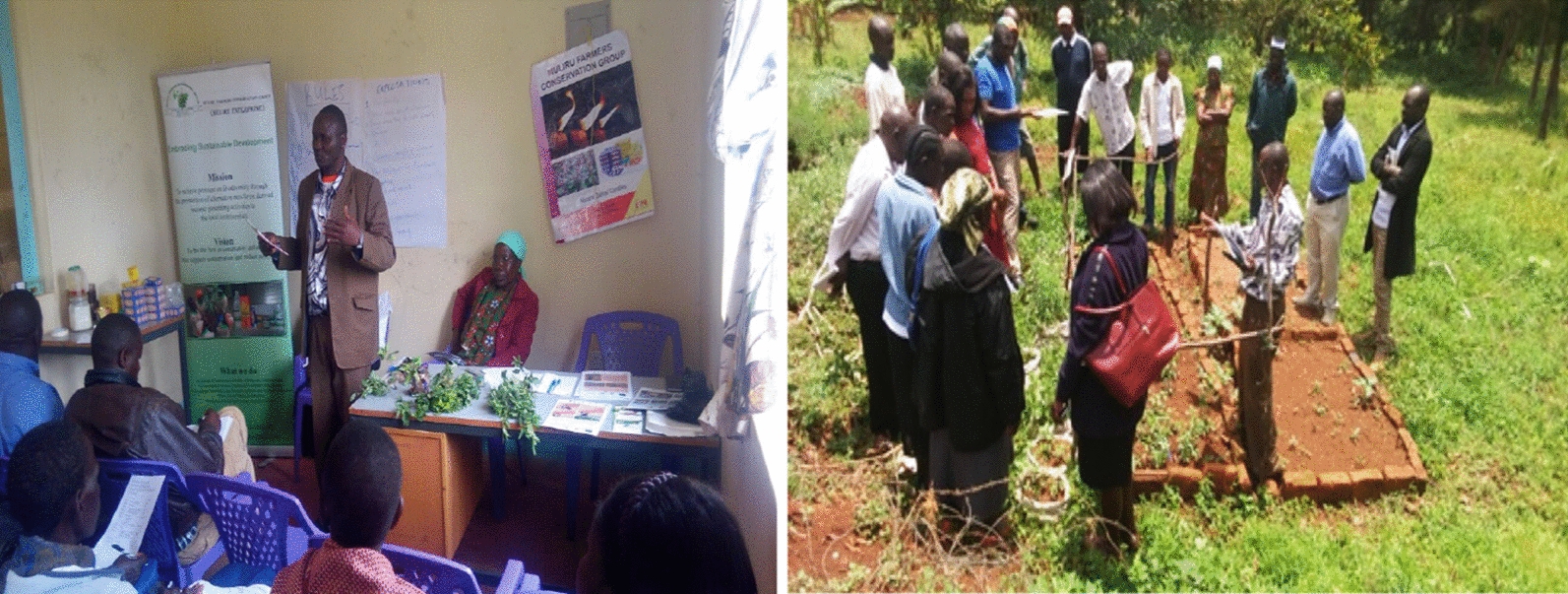
*Ocimum kilimandscharicum* training and field demonstrations taking place in 2017

During the project phase [2016–2018], the community and schools benefited from construction and stocking of fish ponds as part of income generating activities (IGA). Fish farmers received four large community owned fishing nets worth 400 US dollars, courtesy of the county governmentand the fisheries department. Moreover, as part of IVM and IGA, the project also distributed *Ocimum kilimandscharicum* and eucalyptus seedlings to the community. *Ocimum* was introduced to the community because of their medicinal and insecticidal efficacy, their bioactive properties which are used in pharmaceutical, aroma therapeutic and biopesticide industry [46]. On the other hand, eucalyptus seedlings were meant to mop up stagnant water in the water-logged lowland areas as they matured. The planting of eucalyptus trees was also used as part of rehabilitation plan for abandoned brick making pits. Thirty-five members from various local CBOs were randomly selected and trained for one day on *Ocimum* and eucalyptus growing (Fig. 6). Ten CBOs made up of 509 members and four CBOs made up of 152 members received *eucalyptus* and *Ocimum* seedlings respectively as part of IGA. Four primary schools composed of 113 health club members also received eucalyptus seedlings as part of IGA in addition to fish farming. In total, around 20,000 eucalyptus seedlings were distributed to the community as part of IGA initiatives. On average one *eucalyptus* seedlings was selling at 10 Ksh (0.1 US$). Cumulatively, the project was able to reach 25,322 people during its advocacy and social mobilization initiatives (Table 1).

**Table 1:** Summary of collaboration meetings/activities held during the project phase and the number of people reached in each activity, separated by gender

Activity	Female	Male	Total
Community representatives meeting-during project monitoring visits	5	20	25
Meeting with District Social Development Officer (DSDO), Agriculture and Forestry departments	28	33	61
CBO Networking Forums (Child Fund & community leaders meetings)	32	35	67
Stakeholder’s meetings and trainings	84	64	148
IVM Stakeholders Forums in Mbita-Kisumu on 16–17 July 2018. (With County representatives)	15	25	40
Meeting with Nyabondo Hospital CCC (Comprehensive Care Centre) members	62	65	127
Meeting with county Department of Health staffs	82	85	167
Meeting with CBOs representatives	10	30	40
Sensitizations meeting on mass nets distribution (Done by MoH & county government)	25	20	45
Staff planning meetings (at least 2 meetings per month, composed of 5 field staffs on average per meeting)	74	345	419
Community house eaves screening Installation meetings. (180 Houses, Average of 5 People/House)	530	370	900
Farmer’s group field days training/visits	225	350	575
IEC materials distributed- MOCON bulletin (number of Students and farmers benefited)	5094	5576	10,670
Pupils/ Community Members/stakeholders Participation in mosquito day awareness (300 Females & 200 Male pupils participated in drama, health dialogue, role plays and songs)-22/05/2018	1372	1248	2620
Malaria day awareness (Number of people reached)	1000	500	1500
CBOs members benefiting with eucalyptus seedlings distribution	600	409	1009
CBOs Distributed with *Ocimum* seedlings	82	70	152
Schools Health Clubs Distributed with eucalyptus seedlings- 4 schools involved in eucalyptus planting (number of people reached)	58	55	113
Door to door education on Malaria and IVM related activities. (800 Households reached, each averaged, 5 members)	2400	1600	4000
Community/ School Fish Pond stocking, maintenance and harvesting (Number of people reached)	99	2545	2644
Total	11,877	13,445	25,322

## Discussion

Findings from the baseline surveys conducted during the early stage of the project gave pertinent information necessary for planning and improvement of community participation in integrated vector management. The KAP survey on malaria prevention and house screening study conducted in 2018 [39] showed that the community had access to information (91.3%) on malaria prevention and control. Use of mosquito nets was reported to be the most communicated method of malaria prevention. It was also by far the most stated known (97.4%) and applied (97.6%) personal protection method in the area [39]. Such findings about people’s knowledge, attitudes, and practices are important in establishing the actual situation in the community. This information is significant for designing IEC materials and deciding what messages, information and communications tools need to be used [47, 48]. The survey finding also gave information about community perception on the people at risk and vulnerable groups in the community. However, even if people have the understanding of the population at risk of malaria, they may not necessarily take steps to protect themselves and the vulnerable household members [47].

The engagement of communities is a key factor in assuring sustainability and implementation of vector control interventions [49]. Participation may range from adherence to use of intervention strategies such as LLINs, to active involvement in environmental management [50]. The delivery of interventions to the community should be accompanied by a comprehensive and appropriate information, education and communication (IEC) materials and/or behaviour change communication (BCC) campaigns. The IEC and BCC materials should promote community compliance in terms of usage and maintenance of the intervention [13]. On outreach and social mobilization, the community in the project area was sensitized on integrated approaches for malaria control using appropriate information IEC materials such as posters, brochures and fliers [20]. Some of the IEC messages were translated and written into the local language (Dholuo). The translation was meant to enhance awareness on malaria prevention and control in the community especially among the elderly inhabitants and children who would not understand well the English version of the bulletins. These materials were prepared in line with information gathered during the KAP baseline surveys.

Weak advocacy, inadequate promotion of IVM, weak inter-sectoral collaboration and weak coordination networks for vector-borne diseases (VBDs) and lack of information and resources sharing are some of the challenges identified as threatening IVM strategy in sub-Saharan Africa [51, 52]. During the project phase, primary schools were actively involved in social mobilization, outreach and communication for malaria control by participating in various activities like drama, dances, health talks, plays/songs and role plays. The contribution of school children towards dissemination of malaria control messages has been recognized over the years. For example, in Thailand Okabayashi et al. reported that school children disseminated information on malaria to the community through a variety of approaches including issuing newsletters, placing billboards, and holding village events [53]. In Kenya, Onyango-Ouma et al*.* evaluated the potential of school children as health change agents in a rural community in Kenya and observed improved knowledge pertaining to malaria among children and guardians [54]. In Lao PDR, Nonaka et al*.* demonstrated that school children reached out to and influenced women who were not caregivers of target school children to improve their behaviour in relation to malaria control [55]. All these studies have demonstrated that school children are not merely recipients of health education massages, but are also contributors to malaria behavioural change in the community. They play key role in the community as health change agents [56].

Management of VBD control programmes in Africa still remains vertical in nature and targets a single disease in most instances. There is need to explore synergies and maximize the efforts of various sectors in vector control. There is also need for the integration of VBD control interventions by adoption of IVM strategy [35, 52]. During the project, both vertical and horizontal collaboration was enhanced through formal collaboration between health sector and other public and civil sectors. The institutional partnership was forged through collaboration with various government ministries like the Ministry of Agriculture, Fisheries and Forestry Department, the Ministry of Education and Ministry of Health. Collaboration and partnership on malaria control have been reported to have important implications on malaria vector control initiatives globally [18, 57, 58]. For example, engaging the Ministry of Fisheries was essential for promoting improved water management in fish farming and minimize the occurrence of poorly maintained, unproductive or disused fish ponds. Poorly maintained, unproductive or disused fish ponds have been shown to contribute to increased mosquito populations in the area [29, 40, 59]. Collaboration with the Ministry of Education and Fisheries Department enhanced the participation of the 23 local primary schools in extra-curricular activities related to malaria prevention and control as well as in income generating activities.

Capacity building for malaria control is a significant challenge for implementation of IVM strategy in many countries, mainly due to lack of human resources and inadequate infrastructure [35]. The development of human resources for IVM requires a supportive environment, with political and financial commitment for training, recruitment and career paths [17]. In an effort to enhance human resources on the ground, the project in collaboration with the county health department trained a local youth group [MOCON] on house eaves screening. Moreover, 40 CHVs were trained on IVM and malaria case management in the community as part of capacity-building. It was also noted that sustainability of IVM for malaria control would be possible if there is strong social mobilization and political will. The political commitment should be forthcoming if a strong case is to be made for vector-borne diseases and their effect on social-economic development [35]. Politicians are bound to have reservations about changing the existing vector control system, and they have to know how investment in IVM will pay off in terms of health, social and economic benefits and whether IVM can be sustained financially [17]. IVM programmes need to be supported by the government and run through the department of health in collaboration with other stakeholders with an aim of reducing morbidity and mortality due to vector-borne diseases [52, 60]. Additionally, it is important that affected countries put in place relevant national policies and technical strategies to guide IVM implementation for malaria control [61, 62]. Strong advocacy as underscored by the project is, therefore, needed, including packaging information on the burden of vector-borne diseases, their impact on health, the social economic and cultural impacts (e.g. absenteeism from school and work) [62, 63].

Additionally, in partnership with the Department of Fisheries, the project managed to re-train fish farmers. The purpose of training the local people was to give them the necessary knowledge and skills, increase their status and participation, motivate and foster group or team spirit. One of the major stated role of partnerships in IVM is to enhance planning and implementation of agreed upon actions. The partnership helps in decision-making on vector control interventions, sharingr of limited resources and in avoiding duplication of activities [63, 64]. In most developing countries where malaria is endemic, the health sector is underfunded, and funds to support IVM are never readily available. Therefore, strengthening community and stakeholder participation in IVM and intersectoral collaboration and partnership results in reducing duplications, overlaps and saves costs by making better use of existing human and financial resources [20, 52, 60]. IVM could also benefit the health system by increasing the status and motivation of health staff, and improving decision-making abilities and partnerships with other sectors [17, 18, 65]. Careful assessment of the synergies and cost savings brought by IVM partnership should help in gaining sustained support from local authorities, with local allocation of funds in developing countries [20, 29, 49].

## Conclusion

Through advocacy and social mobilization involving educational and income-generating activities, there was improved inter-sectoral collaboration, capacity building and enhanced community participation in IVM. Institutional partnership was also forged through vertical and horizontal collaboration with various government ministries**.** However, more multi-sectoral partnerships and advocacy are needed at national and county government level in order to increase community participation and effectively mobilize resources for IVM.

### Project challenges

Some of the notable challenges faced during the project phase were; unavoidable political interference during 2017 elections that led to national wide strike of health workers. Over expectations and personal interests among some stakeholders were also some of the shortcomings that slowed down local networking and partnerships. The other big challenge was on how to sustain the project activities in the community beyond the donor-funded phase.

### Key lessons learnt

During the project phase, it was noted that sustainable and effective control of malaria is indeed not the sole preserve of the health sector, but requires the collaboration of other sectors. To achieve this, there was need for strong advocacy for promotion of inter and intra-sectoral collaboration in order to effectively and sustainably implement IVM activities. Finally, it was noted that IVM requires strong political will and support in order to succeed, streamline decision-making and share resources. Obtaining the goodwill of local leaders and officials from the health departments was paramount not only for their active participation in the project but also for their role in community mobilization.

## Abbreviations

ACSM: Advocacy, Communication and Social Mobilization
BCC: Behaviour Change Communication
CBO: Community Based Organization
CHVs: Community Health Volunteers
CHWs: Community Health Workers
FGD: Focus Group Discussion
IEC: Information, Education and Communication
IGA: Income Generating Activity
ITNs: Insecticide-treated Nets
IVM: Integrated Vector Management
KEMRI: Kenya Medical Research Institute
KMS: Kenya Malaria Strategy
KNBS: Kenya National Bureau of Statistics
LLINs: Long-lasting insecticidal nets
MOCON: Mosquito Control in Nyabondo
MOH: Ministry of Health
NGO: Non-Governmental Organization
NMCP: National Malaria Control Programme
RBM: Roll Back Malaria Partnership
RDT: Rapid Diagnostic Test
SBCC: Social Behaviour Change Communication
SERU: Scientific and Ethic Review Unit
SERU: Scientific and Ethic Review Unit
TOF: The Organic Farmer
VBD: Vector-Borne Diseases
WHO: World Health Organization
